# Endovascular repair for acute traumatic transection of the descending thoracic aorta: experience of a single centre with a 12-years follow up

**DOI:** 10.1186/s13019-015-0388-5

**Published:** 2015-11-21

**Authors:** Raffaele Serra, Stefano de Franciscis, Raffaele Grande, Lucia Butrico, Paolo Perri, Ciro Indolfi, Pasquale Mastroroberto

**Affiliations:** 1 Interuniversity Center of Phlebolymphology (CIFL). International Research and Educational Program in Clinical and Experimental Biotechnology. Headquarters: University Magna Graecia of Catanzaro, Viale Europa, Catanzaro, 88100 Italy; 2 Department of Medical and Surgical Sciences, University Magna Graecia of Catanzaro, Viale Europa, Catanzaro, Germaneto 88100 Italy; 3 Department of Experimental and Clinical Medicine, University Magna Graecia of Catanzaro, Viale Europa, Catanzaro, 88100 Italy

**Keywords:** Descending thoracic aorta, Endovascular repair, Traumatic transection, TEVAR

## Abstract

**Background:**

Most blunt aortic injuries occur in the proximal proximal descending aorta causing acute transection of this vessel. Generally, surgical repair of the ruptured segment of aorta is associated with high rates of morbidity and mortality and in this view endovascular treatment seems to be a valid and safer alternative. Aim of this article is to review our experience with endovascular approach for the treatment of acute traumatic rupture of descending thoracic aorta.

**Methods:**

From April 2002 to November 2014, 11 patients (9 males and 2 females) were referred to our Department with a diagnosis of acute transection of thoracic aorta. Following preoperative Computed Tomography (CT) evaluation, thoracic endovascular aortic repair (TEVAR) with left subclavian artery coverage was performed. Follow-up consisted clinical and instrumental (CT, Duplex ultrasound) controls at discharge, 1, 3 and 6 months and yearly thereafter.

**Results:**

At 12-year follow up, the overall survival for the entire patients cohort was 100 %, no major or minor neurological complications and no episode of left arm claudication occurred. Cardiovascular, respiratory and bleeding complications, in the early period, was represented by minor, non fatal events. No stent graft failure, collapse, leak or distal migration were detected at CT scan during the entire follow up period.

**Conclusions:**

According to our experience, despite the small number of patient population, TEVAR procedure with with left subclavian artery coverage, performed in emergency settings, seems to provide excellent long term results.

**Trials registration:**

The protocol was registered at a public trials registry, www.clinicaltrials.gov (trial identifier NCT02376998).

## Background

Blunt aortic injury is second only to head injury as the leading cause of death from vehicle crashes as a consequence of deceleration trauma [1–3]. The aortic tear occurs most often at the aortic isthmus, and, in order of frequency, affects the proximal descending aorta, the ascending aorta, the aortic arch, distal descending aorta, and the abdominal aorta [4–6]. This trauma generally progresses into a free rupture of the aorta and causes immediate death at the site of the accident in the 75 % to 80 % of the cases; only 10 % to 15 % of injured people reach a hospital alive [7, 8]. These few patients, in 90 % of cases, have a transection of the thoracic aorta at the isthmus level with a contained rupture [9, 10]. Moreover, these patients often have several other injuries (head trauma, multiple bone fractures, visceral lesions) and for this reason, the immediate treatment of aortic transection can be imperative in decreasing blood loss to prevent continuous fatal bleeding [9]. Several studies have shown that surgical repair of an aortic rupture is associated with high rates of morbidity and mortality, particularly in patients with multiple injuries [11, 12] and it had been delayed, because of coexisting injuries, which rendered the surgical is unacceptably high: severe head trauma, serious skeletal fractures, extensive burns, severe respiratory insufficiency, and sepsis [13–16]. However, the possible benefits of this management strategy are negated when one considers that 2 % to 5 % of these patients develop secondary rupture, mostly within one week of the initial injury [17]. Initially developed for the elective repair of degenerative aneurysms, thoracic endovascular aortic repair (TEVAR) has become the treatment of choice for all conditions, both elective and emergent, involving the descending thoracic aorta [17, 18]. The result has been a decrease in both operative mortality and morbidity for patients with these conditions and it has offered a less invasive and safer alternative to open surgery in acute, high-risk surgical patients [17–19].

The aim of this study is to describe our experience with the endovascular treatment of patients having acute traumatic rupture of the descending thoracic aorta.

## Methods

From April 2002 to November 2014, 11 patients (9 male and 2 female patients) were referred to our department with a diagnosis of acute transection of thoracic aorta. Acute rupture was defined as disruption of the aortic wall with blood flow precariously maintained within the vascular lumen by the adventitia and mediastinal surrounding tissues only (contained rupture) [20]. Signs of impending rupture were considered: discontinuity of aortic contour, contrast media extravasation, rapid growth rate of pseudoaneurysm, periaortic hematoma, and/or hemorrhagic pleural effusion.

This study was approved by the Investigational Review Board, in accordance with the Declaration of Helsinki andthe Guideline for Good Clinical Practice. Before the beginning of the study, all participants provided written informed consent. The protocol was properly registered at a public trials registry, www.clinicaltrial.gov (trial identifier NCT02376998).

### Tevar procedure

As described previously [21], pre-operative evaluation was done by Computed Tomography (CT) considering the following criteria:

1. site of proximal or distal endograft deployment according to the aortic map proposed by Ishimaru [22] and applied by others [23];
2. a minimum length of 15 mm from the aortic lesion, or from the entry site in dissections, to the left subclavian artery and to the coeliac trunk;
3. maximum aortic landing zone diameter of 40 mm;
4. absence of circumferential thrombus or atheroma within the landing zone;
5. absence of significantly tortuous and inadequate access vessels.

According with recent evidences [24, 25], in all cases with a proximal lesion near the origin of the left subclavian artery determining its intentional covering by the stent-graft in order to increase the proximal landing zone, revascularization by supra-aortic transpositions was not considered; patency of both vertebral arteries were documented before the procedure by duplex ultrasonography.

A cerebrospinal fluid catheter (CSF) was inserted before the operation at the level of L3 or L4 to detect neurologic events as spinal cord ischemia due to sustained hypotension during stent-graft placement or to coverage of major medullary arteries, and a pressure of 10 mm Hg or below was maintained. This pressure was monitored for 48 h after the operation in the absence of lower extremity deficits. The mean arterial pressure was kept between 90 and 120 mmHg for the first 72 h to prevent spinal cord hypoperfusion [21].

The Talent™ and, after having been modified, the Valiant™ endoluminal stent-graft systems (Medtronic Inc., Santa Rosa, CA, USA) were used in all patients with its deployment maintaining a systolic pressure at 80 mmHg. The diameter of the stent graft was calculated from the largest diameter of the proximal/distal neck with an oversizing factor of 10-20 %. The procedures were done with local or general anaesthesia in case of unstable pre-operative hemodynamic conditions. During general anesthesia, patients received mechanical ventilation. Blood pressure was monitored by means of right radial artery cannulation. Ceftriaxone (2 g administered intravenously) was administered before the procedure. Depending on the risk of bleeding, a maximum dose of 5000 UI of heparin was administered.

After the procedure was completed, a digital subtraction angiography and echocardiography with color-flow mapping were performed to verify the correct positioning of the stent and to detect any primary endoleak.

Technical success of TEVAR was considered the placement of patent endograft, exclusion of the false lumen in case of Type B aortic dissection (TBAD) and absence of type I or III endoleaks. Endoleaks were defined according to the Committee for Standardized Reporting Practices in Vascular Surgery of The Society for Vascular Surgery/American Association for Vascular Surgery [26]. Type I endoleak was defined as proximal or distal attachment site leak and type III endoleak was considered as junctional leak between stent grafts if more than one graft was used.

Routine examination of heart, lung, liver, and kidney function and contrast-enhanced computed tomographic (CT) scanning or angiographic analysis were conducted in all hemodynamically stable patients. Hemodynamically compromised patients underwent CT analysis, transesophageal echocardiographic (TEE) analysis, or both just before emergency endovascular repair.

### Follow up

Follow-up consisted of CT scan before hospital discharge, at 1, 3 and 6 months, and yearly thereafter. Data from early mortality and postoperative complications as paraplegia or paraparesis, renal and respiratory failure, myocardial infarction, ventricular arrhythmias, congestive heart failure were also collected. We defined paraplegia or paraparesis partial (−paresis) or complete (−plegia) loss of voluntary motor function in the pelvic limbs [27], acute renal failure as acute deterioration of kidney function reflected by a significant increase in serum creatinine [28], respiratory failure as the impaired ability of the respiratory system to maintain adequate oxygen and carbon dioxide homeostasis [29], myocardial infarction as an imbalance between myocardial oxygen supply and demand [30], ventricular arrhythmias (VA) as the presence of ventricular premature beats, ventricular tachycardia (VT), ventricular flutter, torsades de pointes (TdP), accelerated idioventricular rhythm, or ventricular fibrillation (VF) [31, 32], and congestive heart failure when the heart is unable to maintain an adequate circulation of blood in the bodily tissues or to pump out the venous blood returned to it by the veins [33].

To evaluate left arm function a complete clinical and instrumental (duplex ultrasound) evaluation of the left arm was performed in all patients immediately after the procedure and 12, 24, and 72 h later and then at 3,6 and 12 months postoperatively and yearly thereafter, as previously reported [25].

### Statistical analysis

SPSS 21.0 software (IBM) was used for statistical analysis. We defined this study as exploratory; therefore, we did not determine a power calculation. In this light, these results could only be labeled as exploratory.

## Results and discussion

An early emergency endovascular procedure were performed in all patients with a median time from trauma of 3 h (range, 1–10 h). The mean age was 36.9 ± 10.3 years (range, 18–53 years) (Tables 1 and 2). Stent procedures were performed by a multidisciplinary team of cardiovascular surgeons, interventional cardiologists and anesthesiologists and technical success was obtained in all patients submitted to stent graft repair (100 %).

**Table 1:** Baseline of treated patients

Characteristics	
Age, y	
Mean ± SD	36.9 ± 10.3
Median (range)	18-53
Male	9 (81.81 %)
Female	2 (18.18 %)
Hypertension	1
Chronic obstructive pulmonary disease (COPD)	1
Gastrointestinal conditions	0
Paraplegia	0
Congestive heart failure	0
Diabetes	1
Stroke	0
Renal insufficiency	0
Myocardial infarction	1

**Table 2:** Injury characteristics

Characteristic	
Mechanism of blunt aortic injury	
Vehicle accidents	9 (81.81 %)
Pedestrian hit by motor vehicles	1 (9.09 %)
Fall	1 (9.09 %)
Extent of aortic injury	
Grade I: intimal tear	0 (0 %)
Grade II: intramural hematoma	1 (9.09 %)
Grade III: aortic pseudoaneurysm	9 (81.81 %)
Grade IV: free rupture	1 (9.09 %)
Location of aortic injury	
Isthmus (just distal to the left subclavian artery to the third intercostals artery)	11 (100 %)
Distal descending thoracic aorta	0 (0 %)
Associated traumatic injuries	
Lung injury (pneumothorax)	3 (27.27 %)
Unstable fracture cervical spine	1 (9.09 %)
Rib fracture	11 (100 %)
Sternum fracture	2 (18.18 %)
Related injury (solid organ, bowel, bladder, or diaphragm injury)	
Pleural effusion	7 (63.63 %)
Testicle fracture	1 (9.09 %)
Spleen fracture	4 (36.36 %)
Contained rupture of bowel	1 (9.09 %)
Apache –II score	
11-15	2 (18.18 %)
16-20	3 (27.27 %)
26-30	4 (36.36 %)
>30	2 (18.18 %)

### Early outcomes

The early mortality defined as either in-hospital or within 30 days was 0/11 (0 %) (Table 3).

**Table 3:** Early and long term complications

Complication	Early complications	Long term complications
No of patients	11	
Paraplegia/paraparesis	0 (0)	-
Renal failure	0 (0)	-
Respiratory failure	2 (18.2 %)	-
Cardiac arrhythmias		-
Atrial Fibrillation	3 (27.27 %)	
Cerebrovascular accident	0 (0)	-
Bleeding/Pleural effusion	1 (9.1 %)	-
Reintervention	0 (0)	-
Vascular access-related complications		-
Wound dehiscence for infection	1 (9.1 %)	
Left Arm Claudication (clinical or instrumental)	0 (0)	-

After surgical intervention, all patients were admitted to the intensive care unit, where mean stay was 47 ± 15 h. The mean hospital stay was 11 ± 8 days; during this time, no transient or permanent neurologic deficits were reported. No cases of paraplegia/paraparesis, renal failure, cerebrovascular accident, myocardial infarction, ventricular arrhythmias, congestive heart failure were observed. No supra-aortic revascularization as subclavian- to - carotid bypass intervention was performed because no pre-operative hemodinamic alterations of vertebral arteries were revealed by duplex ultrasonography without any case of post-operative cerebrovascular accident.

Two cases of respiratory failure which required prolonged intubation, 1 case of pleural bleeding, 1 case of vascular access complication due to wound dehiscence for infection, 3 cases of Atrial Fibrillation were observed.

A postimplantation syndrome, consisting of leukocytosis and fever, was observed in all patients.

### Long-term outcomes

Overall survival for the entire cohort was 100 %. At 12-year follow-up with a 11 years period of median follow up, all the patients were still alive. None of them required open surgical conversion, or secondary endovascular procedures during follow-up. At CT scan, no stent-graft failure or collapse, leak, or distal migration was detected in any of the 11 survivors.

No episode of left arm claudication (clinical or instrumental) was reported along the entire follow up period (Table 3).

Traditional treatment of blunt aortic injury has been early open surgical repair with graft interposition, with or without adjuncts to maintain distal perfusion. However, open repair carries a 2.9 to 7 % risk of paraplegia and an operative mortality rate ranging from 15 to 23.5 % [34, 35]. Moreover, these patients typically have other severe injuries, and the use of extracorporeal circulation, particularly the use of systemic heparinization, complicates the management of those associated injuries [36]. The introduction of an endoluminal approach represents a significant advance in the care of patients with thoracic aortic transections. Although endovascular management of aortic rupture was initially restricted to high-risk patients with multiple injuries, in many centers it has now become the preferred first treatment even in young or low-risk patients [37, 38]. The benefits of TEVAR include no need for thoracotomy or single lung ventilation, decreased use of systemic anticoagulation, avoidance of aortic cross-clamping, less blood loss, less postoperative pain and lower paraplegia rate and evidences have shown a 7.2 % mortality rate for endovascular repair versus 23.5 % for open repair.

It is known that people who suffer this type of injury and who are treated with TEVAR are young: for this reason, several studies have shown that long-term follow-up data are clearly critical to assess the durability of TEVAR in younger population of patients, who have longer life expectancies than patients with aneurysmal disease. Material failures, such as stent fractures and fabric fatigue, may become more significant during ensuing decades of follow-up. Because the aorta tends to dilate with age, smaller-sized devices appropriate at the time of implantation may lose their fixation over time [39]. Thus, evaluation of long-term device performance in this disease-specific condition is also of high importance. As previously described [39], a 10-year follow-up period in TEVAR patients demonstrated that the reduction in the operative mortality rate of TEVAR, compared with open repair, lasts over time, without any device-related issues. The endoleaks are more frequent in patients with aneurysmal diseases treated with endovascular procedures than patients with aortic transaction: as showed [39], these findings corroborate the observation that aortic expansion seems to be more related to the natural history of the thoracic aorta than to any effect of the stent-graft.

In our study, with a median follow up of 11 years, among the early complications there was no mortality or major neurological complications and no paraplegia/paraparesis events. No patients developed left arm claudication. Cardiovascular, Respiratory and bleeding complications, in the early period, was represented by minor, non-fatal events.

There were no long term complications and the technical success rate was 100 % in all procedures.

The main limitation of this study is the small volume of patients which prevent to make solid conclusion. Furthermore, this study was based upon a non-randomized single center experience.

## Conclusions

According to our experience, and considering also the aforementioned limitiations, we can assume that TEVAR with left subclavian artery coverage for the treatment of acute traumatic rupture of the descending thoracic aorta, can be accomplished safely in emergency settings with minimal morbidity and excellent long term results. Nevertheless, further studies with a wider population of patients are required to confirm our results.

## Abbreviations

CSF: cerebrospinal fluid catheter
CT: computed tomography
TBAD: type B aortic dissection
TEVAR: thoracic endovascular aortic repair
TdP: torsades de pointes
TEE: transesophageal echocardiography
VF: ventricular fibrillation
VT: ventricular tachycardia
